# The Bcl-2/xL inhibitor ABT-263 increases the stability of Mcl-1 mRNA and protein in hepatocellular carcinoma cells

**DOI:** 10.1186/1476-4598-13-98

**Published:** 2014-04-30

**Authors:** Bin Wang, Zhenhong Ni, Xufang Dai, Liyan Qin, Xinzhe Li, Liang Xu, Jiqin Lian, Fengtian He

**Affiliations:** 1 Department of Biochemistry and Molecular Biology, College of Basic Medical Sciences, Third Military Medical University, 30 Gaotanyan, Chongqing 400038, China; 2 Department of Educational Science College, Chongqing Normal University, Chongqing 400038, China; 3 Departments of Molecular Biosciences and Radiation Oncology, University of Kansas Cancer Center, University of Kansas, Lawrence 66045-7534, USA

**Keywords:** ABT-263, Mcl-1, Stability, HCC

## Abstract

**Background:**

Hepatocellular carcinoma (HCC) is one of the major causes of mortality. ABT-263 is a newly synthesized, orally available Bcl-2/xL inhibitor that shows promising efficacy in HCC therapy. ABT-263 inhibits the anti-apoptotic activity of Bcl-2 and Bcl-xL, but not Mcl-1. Previous reports have shown that ABT-263 upregulates Mcl-1 in various cancer cells, which contributes to ABT-263 resistance in cancer therapy. However, the associated mechanisms are not well known.

**Methods:**

Western blot, RNAi and CCK-8 assays were used to investigate the relationship between Mcl-1 upregulation and ABT-263 sensitivity in HCC cells. Real-time PCR and Western blot were used to detect Mcl-1 mRNA and protein levels. Luciferase reporter assay and RNA synthesis inhibition assay were adopted to analyze the mechanism of Mcl-1 mRNA upregulation. Western blot and the inhibition assays for protein synthesis and proteasome were used to explore the mechanisms of ABT-263-enhanced Mcl-1 protein stability. Trypan blue exclusion assay and flow cytometry were used to examine cell death and apoptosis.

**Results:**

ABT-263 upregulated Mcl-1 mRNA and protein levels in HCC cells, which contributes to ABT-263 resistance. ABT-263 increased the mRNA level of Mcl-1 in HCC cells by enhancing the mRNA stability without influencing its transcription. Furthermore, ABT-263 increased the protein stability of Mcl-1 through promoting ERK- and JNK-induced phosphorylation of Mcl-1^Thr163^ and increasing the Akt-mediated inactivation of GSK-3β. Additionally, the inhibitors of ERK, JNK or Akt sensitized ABT-263-induced apoptosis in HCC cells.

**Conclusions:**

ABT-263 increases Mcl-1 stability at both mRNA and protein levels in HCC cells. Inhibition of ERK, JNK or Akt activity sensitizes ABT-263-induced apoptosis. This study may provide novel insights into the Bcl-2-targeted cancer therapeutics.

## Background

Hepatocellular carcinoma (HCC) is one of the major causes of mortality in developing countries, such as in China, and its prevalence ranks the fifth of all tumors with rapid increasing morbidity [1]. Currently, the efficacy of traditional chemotherapy for HCC is often unsatisfied [2]. Therefore, it is of great priority to develop novel molecular targeted compounds. Recent studies have shown that the inhibitors of Bcl-2 exhibit promising antitumor activity [3].

Bcl-2 family consists of three categories of proteins, namely anti-apoptotic members, apoptosis executors and pro-apoptotic BH3-only proteins. The balance of these proteins contributes to survival and homeostasis of both normal and tumor cells [4]. However, overexpression of anti-apoptotic members Bcl-2 and Bcl-xL always happens in tumors and indicates a poor prognosis [5-8]. Meanwhile, previous reports have also shown that the levels of Bcl-2/xL are closely related to the pathological grade and survival rate of HCC [9,10]. These studies imply that Bcl-2/xL may serve as potential therapeutic targets for HCC.

Some of the Bcl-2 inhibitors (that have been) developed are a group of natural or synthesized compounds that target anti-apoptotic Bcl-2 family members especially Bcl-2 and Bcl-xL. ABT-263, also known as Navitoclax, is an orally available analog of ABT-737, which can bind to Bcl-2 and Bcl-xL, but not Mcl-1 [4]. Several studies have shown that ABT-263 exerts optimistic anti-tumor effects, especially in haematological malignancies and non-small cell lung cancer [11]. Furthermore, ABT-263 is now in phaseII clinical trials for several types of tumor with initial results [12,13]. However, previous studies have shown that ABT-263 upregulates Mcl-1 protein, which ultimately contributes to drug resistance [14,15].

Mcl-1 is an important anti-apoptotic protein that mainly distributes in mitochondria and cytoplasm. Mcl-1 exerts anti-apoptotic effects by interacting with pro-apoptotic proteins such as Bim, Noxa, Bak and Bax. Also, Mcl-1 may function by facilitating normal mitochondrial fusion, ATP production and respiration [16]. Therefore, Mcl-1 protein level is elaborately regulated in both normal and tumor cells [17], among which phosphorylation modification is a quite significant way. Others reporting results and our previous data have shown that ABT-263 upregulates Mcl-1 in HCC cells, which is the crucial reason for ABT-263 resistance in cancer therapy. However, the associated mechanisms are not well known [14,18]. In the present study, we for the first time demonstrated that ABT-263 upregulated Mcl-1 by enhancing the stability of both Mcl-1 mRNA and protein, which contributed to ABT-263 resistance in HCC cells. Moreover, inhibition of ERK, JNK or Akt activity sensitized ABT-263-induced apoptosis. This study may provide novel insights into the Bcl-2-targeted cancer therapeutics.

## Results

### Upregulation of Mcl-1 is correlated with ABT-263 resistance in HCC cells

**Figure 1:**
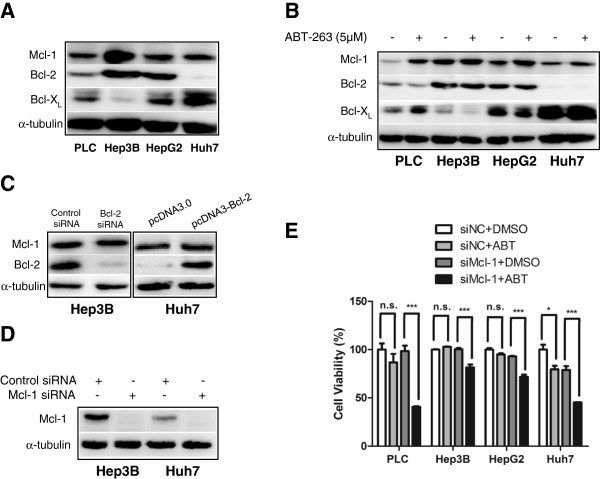
**Upregulation of Mcl-1 is correlated with ABT-263 resistance in HCC cells. (A)** The expression profiles of Bcl-2, Bcl-xL and Mcl-1 were analyzed by Western blot in four HCC cell lines, taking α-tubulin as a loading control. **(B)** HCC cells were treated with 5 μM ABT-263 for 18 h, then the expression levels of Mcl-1, Bcl-2 and Bcl-xL were analyzed by Western blot. **(C)** Hep3B and Huh7 cells were transfected with either Bcl-2 siRNA (or control siRNA) or pcDNA3-Bcl-2 plasmid (or control plasmid pcDNA3.0) for 36 h, then the level of Mcl-1 was analyzed by Western blot. **(D)** Hep3B and Huh7 cells were transfected with control siRNA or Mcl-1 siRNA for 36 h, then Mcl-1 protein level was analyzed by Western blot. **(E)** After transfected with Mcl-1 siRNA or control siRNA for 24 h, the HCC cells were treated with 5 μM ABT-263 or vehicle DMSO for another 36 h. Then the cell viability was analyzed by CCK-8 kit. Data were expressed as the mean ± SD from three independent experiments. (*: *P* < 0.05; ***: *P* < 0.0001).

Firstly, the expression levels of anti-apoptotic Bcl-2 family members Bcl-2, Bcl-xL and Mcl-1 were analyzed with Western blot in HCC cell lines PLC/PRF/5, Hep3B, HepG2 and Huh7. As shown in Figure 1A, Mcl-1 was highly expressed in all four HCC cell lines, but the levels of Bcl-2 and Bcl-xL differed. Hep3B cells had low level of Bcl-xL and Huh7 cells had almost no detectable Bcl-2. Upon treatment with ABT-263, the level of Mcl-1 increased dramatically in all HCC cell lines, but the levels of Bcl-2 and Bcl-xL did not change significantly (Figure 1B). Another Bcl-2 inhibitor AT-101 had similar effect on Mcl-1 expression in HCC cells (Additional file 1: Figure S1). To test whether the upregulation of Mcl-1 is affected by Bcl-2 level, we knocked down Bcl-2 in Hep3B cells and overexpressed it in Huh7 cells, respectively. As shown in Figure 1C, the level of Mcl-1 remained unchanged upon Bcl-2 downregulation or overexpression. Similar results were also found when Bcl-xL was knocked down in Huh7 cells or overexpressed in Hep3B cells (date not shown). These results indicated that ABT-263-induced Mcl-1 upregulation was independent of the levels of Bcl-2/xL in HCC cells. Furthermore, consistent with previous reports [19-21], Mcl-1 knockdown significantly enhanced the cytotoxicity of ABT-263 in HCC cells (Figure 1D and E). The above data indicated that the drug resistance of ABT-263 was, at least partially, mediated by Mcl-1 upregulation, which was not associated with the expression levels of Bcl-2/xL in HCC cells.

### ABT-263 upregulates Mcl-1 at both mRNA and protein levels

**Figure 2:**
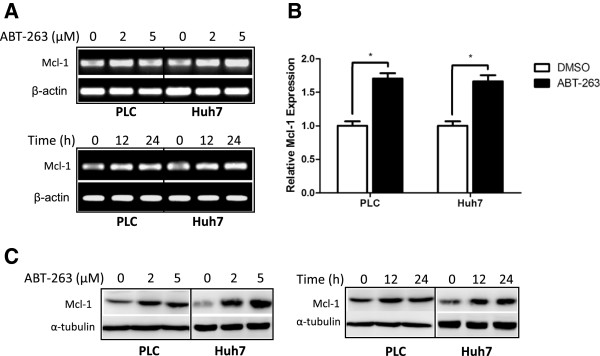
**ABT-263 upregulates Mcl-1 at both mRNA and protein levels. (A)** PLC and Huh7 cells were treated with various doses of ABT-263 for 24 h or with 5 μM ABT-263 for indicated times. Then RT-PCR was performed to detect the level of Mcl-1 mRNA, taking β-actin as a control. **(B)** HCC cells were treated with 5 μM ABT-263 or vehicle DMSO for 24 h, then qPCR was performed to quantitate Mcl-1 mRNA, taking β-actin as a control. Data were represented as mean ± SD from three independent experiments. (*: *P* < 0.05). **(C)** HCC cells were treated as described in **(A)**, then the level of Mcl-1 was analyzed by Western blot, taking α-tubulin as a loading control.

To investigate the underlying mechanism of ABT-263-induced Mcl-1 upregulation in HCC cells, both mRNA and protein levels of Mcl-1 were analyzed after treatment with ABT-263. Since PLC and Huh7 cell lines had a higher sensitivity to ABT-263 after Mcl-1 knockdown (Figure 1E), they were chosen as target cells. As shown in Figure 2, ABT-263 upregulated Mcl-1 at both mRNA and protein levels in PLC and Huh7 cells revealed by RT-PCR (Figure 2A), real-time PCR (Figure 2B) and Western blot (Figure 2C).

### ABT-263 increases the mRNA stability of Mcl-1

**Figure 3:**
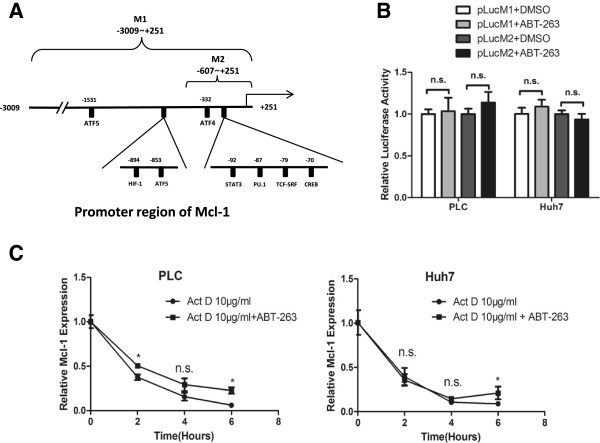
**ABT-263 increases the mRNA stability of Mcl-1. (A)** Schematic representation of Mcl-1 promoter region containing several transcription factor binding sites. Two fragments (−3009 to +251 and −607 to +251) were separately amplified from genomic DNA of HepG2 cells and inserted into pGL3-basic vector, and the resulting plasmids were named as pLucM1 and pLucM2 respectively. **(B)** After co-transfected with pLucM1 or pLucM2 and pCMV-β-gal plasmid for 12 h, the HCC cells were treated with 5 μM ABT-263 or vehicle DMSO for another 24 h. Then the luciferase assay was performed. Data were expressed as mean ± SD from three independent experiments. **(C)** HCC cells were treated with 10 μg/ml actinomycin D (Act D) in the presence or absence of 5 μM ABT-263 for indicated times, then the level of Mcl-1 mRNA was quantified by qPCR, taking β-actin as a control. Data were represented as mean ± SD from three independent experiments. (*: *P* < 0.05).

To figure out the mechanisms of ABT-263-mediated Mcl-1 mRNA upregulation, the promoter region of Mcl-1 gene (−3009 to +251, named M1) was cloned into reporter vector pGL3-basic, and the resulting plasmid was named as pLucM1 (Figure 3A). Meanwhile, the promoter region (−607 to +251, named M2) containing the binding sites for several predicted transcriptional factors was also cloned into pGL3-basic, and the resulting plasmid was named as pLucM2 (Figure 3A). Then PLC and Huh7 cells were separately transfected with pLucM1 and pLucM2 and followed by the treatment with ABT-263. As shown in Figure 3B, ABT-263 didn’t affect the activity of Mcl-1 promoter in HCC cells, neither in pLucM1 nor in pLucM2. Subsequently, PLC and Huh7 cells were treated with transcription inhibitor actinomycin D (Act D) in the presence or absence of ABT-263. As shown in Figure 3C, ABT-263 co-treatment significantly enhanced the mRNA stability of Mcl-1 compared to Act D treatment alone (*p* < 0.05). These results indicated that ABT-263 upregulated Mcl-1 mRNA level via increasing the mRNA stability instead of activating its transcription in HCC cells.

### ABT-263 increases the protein stability of Mcl-1

**Figure 4:**
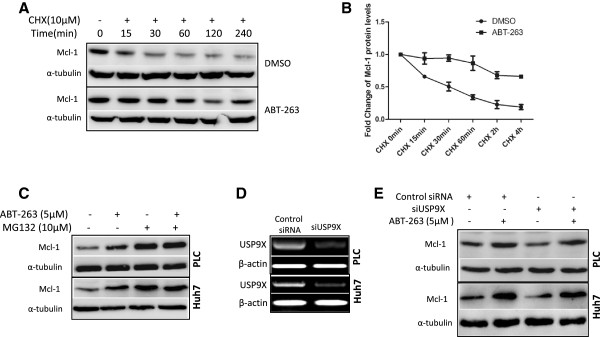
**ABT-263 increases the protein stability of Mcl-1. (A, B)** After pretreated with 5 μM ABT-263 or vehicle DMSO for 12 h, PLC cells were treated with 10 μM CHX for indicated times. The level of Mcl-1 protein was detected by Western blot taking α-tubulin as a loading control **(A)**, and the relative level of Mcl-1 protein was calculated **(B)** as follows: (1) quantifying the mean ratio of Mcl-1/α-tubulin for each sample according to the Western blot results from triplicate assays and (2) calculating the fold induction of Mcl-1 by normalizing the mean Mcl-1/α-tubulin ratio of the tested sample to that of corresponding control (CHX 0 min). Data were expressed as mean ± SD from three independent experiments. **(C)** After pretreated with 10 μM proteasome inhibitor MG132 for 2 h, the HCC cells were incubated with 5 μM ABT-263 or vehicle DMSO for 6 h. Then the level of Mcl-1 protein was detected by Western blot. **(D)** HCC cells were transfected with USP9X-specific siRNA or control siRNA for 36 h, then the mRNA level of USP9X was analyzed by RT-PCR. **(E)** After transfected with USP9X-specific siRNA or control siRNA for 24 h, the HCC cells were treated with 5 μM ABT-263 or vehicle DMSO for another 18 h. Then the level of Mcl-1 protein was analyzed by Western blot.

To assess whether the increase of Mcl-1 protein solely results from the upregulation of Mcl-1 mRNA, the HCC cells were treated with translation inhibitor cycloheximide (CHX). As shown in Figure 4A and B, the level of Mcl-1 protein decreased dramatically after treatment with CHX alone, and the half-life of Mcl-1 protein was 30 min. Co-treatment with ABT-263 and CHX markedly attenuated the degradation of Mcl-1 protein, and the half-life of Mcl-1 protein reached to more than 4 h. These results indicated that ABT-263 enhanced Mcl-1 protein stabilization in HCC cells. Meanwhile, ABT-263 could not further upregulate Mcl-1 protein level after proteasome was inhibited by MG132, suggesting that ABT-263 might upregulate Mcl-1 protein level by decreasing proteasome-mediated degradation (Figure 4C). As to whether ABT-263 affected the ubiquitination-mediated Mcl-1 degradation, the role of deubiquitinase USP9X (ubiquitin-specific peptidase 9, X-linked) was investigated. As shown in Figure 4D and E, knockdown of USP9X didn’t affect ABT-263-mediated Mcl-1 accumulation, indicating that USP9X-mediated deubiquitination doesn’t contribute to ABT-263-enhanced Mcl-1 stability.

### Activation of ERK and JNK involves in ABT-263-induced stabilization of Mcl-1 protein

**Figure 5:**
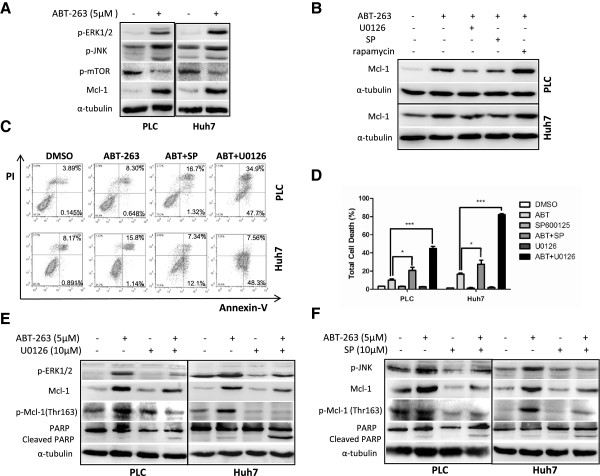
**ABT-263 enhances ERK- and JNK-mediated Mcl-1**^**Thr163 **^**phosphorylation and protein stabilization. (A)** HCC cells were treated with 5 μM ABT-263 for 18 h, and then the phosphorylation of ERK1/2, JNK and mTOR were detected by Western blot, taking α-tubulin as a loading control. **(B)** After pretreated with 10 μM ERK1/2 inhibitor U0126, 10 μM JNK inhibitor SP600125 (SP), or 2 μM mTOR inhibitor rapamycin for 2 h, the HCC cells were treated with 5 μM ABT-263 for another 18 h. Then the level of Mcl-1 protein was analyzed by Western blot. **(C)** After pretreated with 10 μM U0126 or 10 μM SP600125 for 2 h, the HCC cells were treated with 5 μM ABT-263 for 24 h, then the cells were stained with annexin V-FITC/PI and analyzed by flow cytometry. **(D)** After pretreated with 10 μM U0126 or 10 μM SP600125 for 2 h, the HCC cells were treated with 5 μM ABT-263 for another 24 h. Then the total cell death was calculated by trypan blue exclusion assay. Data were expressed as mean ± SD from three independent experiments. (*: *P* < 0.05, ***: *P* < 0.0001). **(E, F)** After pretreated with 10 μM U0126 or 10 μM SP600125 for 2 h, the HCC cells were treated with 5 μM ABT-263 for another 18 h. Then PARP and cleaved PARP, p-ERK, p-JNK, Mcl-1 and p-Mcl-1(Thr163) were analyzed by Western blot.

It is known that there is a unique PEST region in Mcl-1 protein and the phosphorylation of this region is closely associated with Mcl-1 protein stability [22], so we analyzed the activity of several kinases which directly phosphorylate Mcl-1, including extracellular regulated kinase (ERK) and c-Jun terminal kinase (JNK). Meanwhile, phosphorylation of mammalian target of rapamycin (mTOR) was also detected upon ABT-263 treatment since its activation through phosphorylation can regulate the translational process of Mcl-1 protein [23]. As shown in Figure 5A, ERK and JNK were activated while mTOR was repressed after treatment with ABT-263. To further clarify the role of these kinases in ABT-263-enhanced Mcl-1 protein stabilization, their inhibitors were used. ERK inhibitor U0126 and JNK inhibitor SP600125, but not mTOR inhibitor rapamycin, markedly attenuated ABT-263-caused Mcl-1 upregulation (Figure 5B). Moreover, ERK and JNK inhibitors significantly increased ABT-263-induced apoptosis in PLC and Huh7 cells revealed by annexin V-FITC/PI staining flow cytometry analysis (Figure 5C), trypan blue exclusion assay (Figure 5D) and Western blot for enhanced PARP cleavage (Figure 5E and F). These results indicated that activation of ERK and JNK, but not mTOR, involved in ABT-263-mediated Mcl-1 protein stabilization and drug resistance.

### ABT-263 enhances ERK- and JNK-mediated Mcl-1^Thr163^ phosphorylation

To further investigate the concrete mechanisms of ERK- and JNK-mediated Mcl-1 stabilization, the phosphorylation status of Mcl-1^Thr163^ was analyzed. As shown in Figure 5E and F, inhibition of ERK or JNK significantly attenuated ABT-263-induced Mcl-1^Thr163^ phosphorylation and Mcl-1 accumulation, suggesting that the phosphorylation of Mcl-1^Thr163^ may contribute to ERK- and JNK-mediated Mcl-1 stabilization upon ABT-263 treatment in HCC cells.

### Akt-mediated GSK-3β inactivation also involves in ABT-263-induced Mcl-1 stabilization

**Figure 6:**
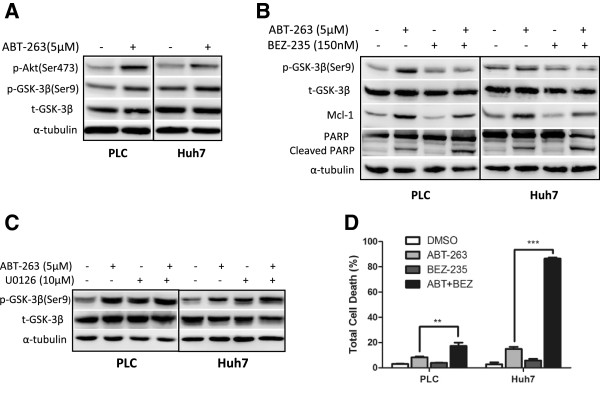
**Akt-mediated GSK-3**β **inactivation also involves in ABT-263-induced stabilization of Mcl-1 protein. (A)** HCC cells were treated with 5 μM ABT-263 for 18 h, then p-Akt (Ser473), p-GSK3β (Ser9) and total GSK3β (t-GSK3β) were detected by Western blot, taking α-tubulin as a loading control. **(B)** After pretreated with 150nM PI3K/mTOR dual inhibitor NVP-BEZ235 for 2 h, the HCC cells were treated with 5 μM ABT-263 for 18 h. Then p-GSK3β (Ser9) and t-GSK3β, Mcl-1, PARP and cleaved PARP were analyzed by Western blot. **(C)** After pretreated with 10 μM ERK1/2 inhibitor U0126 for 2 h, the HCC cells were treated with 5 μM ABT-263 for 18 h. Then p-GSK3β (Ser9) and t-GSK3β were detected by Western blot. **(D)** After pretreated with 150nM NVP-BEZ235 for 2 h, the HCC cells were treated with 5 μM ABT-263 for another 24 h. Then the total cell death was analyzed by trypan blue exclusion assay. Data were expressed as mean ± SD from three independent experiments. (**: *P* < 0.01, ***: *P* < 0.0001).

Since Serine^159^ is also closely related with Mcl-1 stability and this site is mainly phosphorylated by GSK-3β [24], we tested whether GSK-3β involves in ABT-263-induced Mcl-1 stabilization in HCC cells. As shown in Figure 6A, ABT-263 increased the phosphorylation of GSK-3β, but no effect on total GSK-3β. Meanwhile, ABT-263 enhanced the phosphorylation of Akt, an upstream signal molecule of GSK-3β. Suppression of Akt by its inhibitor BEZ-235 dramatically attenuated ABT-263-mediated GSK-3β phosphorylation, Mcl-1 upregulation and apoptosis resistance (Figure 6B). Subsequently, we checked whether the phosphorylation of GSK-3β is also affected by ERK, another upstream regulator of GSK-3β. As shown in Figure 6C, inhibition of ERK with U0126 had no effect on ABT-263-triggered GSK-3β phosphorylation, indicating that GSK-3β activity was not regulated by ERK in this process. Furthermore, Akt inhibitor also increased the cytotoxicity of ABT-263 in HCC cells (Figure 6D). These results indicated that Akt-mediated GSK-3β inactivation also involves in ABT-263-induced Mcl-1 stabilization, possibly through regulating the phosphorylation of Mcl-1^Ser159^.

## Discussion

**Figure 7:**
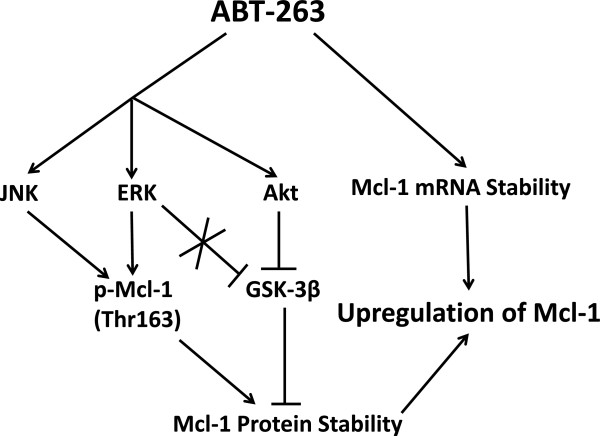
**Schematic illustration for the action mechanisms of ABT-263-induced Mcl-1 upregulation in HCC cells.** ABT-263 increases Mcl-1 mRNA level by augmenting its stability instead of transcriptional activation. Meanwhile, ABT-263 enhances Mcl-1 protein stability by regulating the phosphorylation status of Mcl-1. ERK- and JNK-mediated Mcl-1^Thr163^ phosphorylation contribute to ABT-263-induced Mcl-1 protein stability. Akt-mediated GSK-3β inactivation also plays important role in preventing Mcl-1 protein degradation in the presence of ABT-263. By increasing Mcl-1 stability at both mRNA and protein levels, ABT-263 induces Mcl-1 upregulation.

In the present study, we demonstrated that ABT-263 upregulated Mcl-1 by increasing the stability of Mcl-1 mRNA and protein in HCC cells. As shown in the working model (Figure 7), ABT-263 increased Mcl-1 mRNA level by augmenting its stability instead of transcriptional activation. Meanwhile, ABT-263 enhanced Mcl-1 protein stability by regulating the phosphorylation status of Mcl-1. ERK- and JNK-mediated Mcl-1^Thr163^ phosphorylation contributed to ABT-263-induced Mcl-1 protein stability. Akt-mediated GSK-3β inactivation also played important role in preventing Mcl-1 protein degradation in the presence of ABT-263.

ABT-263, a newly-developed, oral-tolerant Bcl-2/xL inhibitor, has shown promising anti-tumor efficacy in non-small cell lung cancer and acute lymphoblastic leukemia as single agent both *in vitro* and *in vivo*[25]. Meanwhile, ABT-263 can markedly sensitize several clinical drugs in cancer therapy [26,27]. However, a recent study has demonstrated that HCC cells are relatively resistant to ABT-737 (analog of ABT-263) compared to leukemia and lung carcinomas [28]. Furthermore, it has been indicated that ABT-737-induced Mcl-1 upregulation contributes to this resistance [14]. Consistent with ABT-737, our results showed that both ABT-263 and another Bcl-2 inhibitor AT-101 upregulated Mcl-1 in HCC cells, which at last resulted in drug resistance. So it is important to clarify the associated mechanisms of ABT-263-induced Mcl-1 upregulation in HCC cells.

It is known that Mcl-1 is an important anti-apoptotic protein, which is now becoming a quite important target for cancer therapy [29]. Characteristically, it has a short half-life and is elaborately regulated at different levels [17]. We found that ABT-263 increased Mcl-1 mRNA level in HCC cells. It is also reported that Mcl-1 can be regulated by several transcription factors, including STAT3 [30], ATF4 [31], CREB [32] and HIF-1 [33]. However, the luciferase assay results in this study demonstrated that ABT-263 did not increase the transcriptional activity of Mcl-1 promoter, indicating that these transcription factors may not play dominated roles in this process. Furthermore, we demonstrated that ABT-263 enhanced Mcl-1 mRNA stability in HCC cells. It is known that RNA stability is affected by various factors such as RNases and RNA binding proteins, but just only one RNA binding protein CUGBP2 has been reported to play a role in Mcl-1 mRNA stabilization [34]. Therefore, it is unclear at present whether ABT-263-enhanced Mcl-1 mRNA stability is associated with CUGBP2, which is interesting and needs further studies.

Besides mRNA level, protein stability also plays important role in the upregulation of Mcl-1 protein. It is known that the phosphorylation of Mcl-1 is closely associated with Mcl-1 protein stabilization [22]. Serine^159^ and Threonine^163^ are two important phosphorylation sites in Mcl-1 PEST region to determine the fate of Mcl-1 degradation. Mcl-1 can be phosphorylated by ERK at its Thr^163^ site, which prolongs the half life of this protein [35]. ERK mediated-phosphorylation at Thr^163^ represents an important resistant mechanism in leukemia cells [15] and the inhibition of MEK/ERK sensitizes the anti-tumor effect of ABT-737 [36]. Consistent with these reports, our study showed that ERK-mediated Thr^163^ phosphorylation of Mcl-1 contributed to ABT-263 resistance in HCC cells. JNK, another important member of MAPK family, can phosphorylate Mcl-1 at several sites, but the effect of JNK on Mcl-1 is varied [22]. JNK-mediated Thr^163^ phosphorylation may lead to enhanced Mcl-1 degradation [37] or increased Mcl-1 stabilization [38]. Our data demonstrated that ABT-263 increased JNK-mediated Mcl-1^Thr163^ phosphorylation, which enhanced Mcl-1 protein stability in HCC cells. Furthermore, both ERK and JNK inhibitors sensitized ABT-263-induced apoptosis and cell death by downregulating Mcl-1 in HCC cells, which may be novel ways to sensitize ABT-263 in HCC therapy.

GSK-3β plays an important role in glucose metabolism in mammalian cells. After being phosphorylated at Serine^9^, GSK-3β loses its activity. It is known that Mcl-1 can be phosphorylated by GSK-3β at Ser^159^ site, which decreases Mcl-1 stability [24]. A recent study has shown that ABT-263 enhances the anti-tumor effect of PI3K inhibitor in GSK3-dependent manner in human myeloid leukemia cells, but the detailed mechanisms are still not clear [39]. Our study demonstrated that ABT-263 promoted GSK-3β inactivation and Mcl-1 stability via Akt pathway, indicating that inhibition of Akt may be a good strategy to sensitize ABT-263 in HCC treatment.

It is well known that Bcl-2/xL are involved in regulating the homeostasis of apoptosis, autophagy and oxidative stress in the cells [40], which are associated with ERK, JNK and Akt pathways. ABT-263 is known as a specific inhibitor of Bcl-2/xL, so the mechanisms by which ABT-263 activates ERK, JNK and Akt may be complicated. Our previous data have shown that Bcl-2 inhibitor apogossypolone can induce reactive oxygen species (ROS) in HCC cells, which results in the activation of multiple vital signaling pathways including ERK, JNK and Akt pathways [41]. In the present study, we demonstrated that ABT-263 could induce the phosphorylations of ERK, JNK and Akt, which were markedly attenuated by the widely used antioxidant N-acetyl-cysteine (Additional file 1: Figure S2), suggesting that ABT-263 may activates ERK, JNK and Akt via , at least partially, inducing ROS production.

## Conclusions

In conclusion, our study demonstrates that ABT-263 upregulates Mcl-1 through increasing its mRNA and protein stability, which contributes to the resistance of ABT-263 in HCC cells. Inhibition of ERK-, JNK- or Akt-mediated Mcl-1 stability may confer Bcl-2 inhibitor better anti-tumor effect in HCC cells. Our results may provide more details to Bcl-2-targeted therapeutics and give insights into the future clinical trials of Bcl-2 inhibitors in HCC therapy.

## Materials and methods

### Materials

The cell culture reagents were purchased from Hyclone (Waltham, MA, USA). ABT-263, cycloheximide, SP600125, rapamycin, NVP-BEZ235 and N-acetyl-cysteine were purchased from Sigma-Aldrich (Louis, MO, USA). U0126, Act D, MG132, the antibody against α-tubulin, BCA protein assay kit and RIPA lysis buffer were purchased from Beyotime Biotechnology (Shanghai, China). AnnexinV-FITC/propidium iodide (PI) apoptosis detection kit was purchased from BD bioscience (BD, NJ, USA). Cell Counting Kit-8 (CCK-8) was from Dojindo (Shanghai, China). Trizol agent, M-MLV transcriptase and Lipofectamin 2000 were from Invitrogen (Carlsbad, CA, USA). SYBR qPCR master mix, PrimeSTAR HS DNA polymerase, restriction endonuclease *Nhe*Iand *Hin*dIII were from TAKARA (Shiga, Japan). pGL3-basic vector, pCMV-β-gal plasmid, luciferase assay and β-gal assay systems were from Promega (Madison, WI, USA). Antibodies of Mcl-1 and Bcl-2 were purchased from Santa Cruz Biotechnology (Santa Cruz, CA, USA). Antibodies separately against Bcl-xL, PARP, phosphorylated ERK1/2 (p-ERK1/2, Thr202/Tyr204), total-ERK, p-JNK(Thr183/Tyr185), p-mTOR(Ser2448), p-Mcl-1(Thr163), p-Akt(Ser473), p-GSK-3β(Ser9) and total-GSK-3β were from Cell Signaling Technology (Boston, MA, USA). HRP-conjugated goat anti-rabbit and anti-mouse IgG were purchased from Zhongshan Company (Beijing, China). siRNAs to Bcl-2, Bcl-xL, USP9X and control siRNAs were from Dharmacon (Lafayette, CO, USA). pcDNA3-Bcl-2 and pcDNA3-Bcl-xL expression plasmids were kindly gifts from University of Michigan( pcDNA3.0 was used as negative control).

### Cell culture

Human HCC cell lines PLC/PRF/5, HepG2, Huh7 and Hep3B were purchased from American Type Culture Collection (ATCC), and cultured in high-glucose DMEM (Dulbecco’s modified Eagle’s medium) with 10% FBS (fetal bovine serum), streptomycin (100 μg/mL) and penicillin(100 U/mL). These cell lines were originally tested by ATCC and passaged less than 6 months in the lab.

### Quantitative polymerase chain reaction (qPCR)

After treatment, the cells were lysed and total RNA was extracted with Trizol agent as described [42], and first-strand cDNA was synthesized using M-MLV transcriptase. qPCR was performed to detect the level of Mcl-1 mRNA using SYBR qPCR master mix in a 25 μl volume according to the manufacturer’s instruction. The sequences of different primers were as follows: Mcl-1: forward primer 5′-AAAGCCTGTCTGCCAAAT-3′ and reverse primer 5′-CCTATAAACCCACCACTC-3′; USP9X: forward primer 5′-CCTGCTGGTGCACCTCTGGC-3′ and reverse primer 5′-AGGCCGGTGTCCCATGCAA-3′; β-actin: forward primer 5′-ATCGTGCGTGACATTAAGGAGAAG-3′ and reverse primer 5′-AGGAAGGAAGGCTGGAAGAGTG-3′.

### Western blot

After treatment, the cells were harvested and whole-cell lysates were prepared. The protein concentrations were measured by BCA protein assay kit. Subsequently, Western blot analysis was performed as described [41].

### Transfection of siRNA and Bcl-2/xL expression plasmid

The HCC cells were separately transfected with siRNAs to Bcl-2 (or Bcl-xL or USP9X) and control siRNA using Lipofectamine 2000 according to the manufacturer’s instruction. Similarly, the expression plasmid pcDNA3-Bcl-2 or pcDNA3-Bcl-xL was transfected into the corresponding HCC cells, taking pcDNA3.0 as negative control.

### Cell viability assay

Cell viability assay was performed by using Cell Counting Kit-8 (CCK-8). Briefly, cells were seeded in triplicate in 96-well plates and given different treatments for indicated time, then the OD value at 450 nm was detected according to the manufacturer’s instruction.

### Plasmid construction

Human Mcl-1 promoter regions −3009 to +251(M1) and −607 to +251(M2) were amplified by PCR using PrimeSTAR HS DNA polymerase taking genomic DNA of HepG2 cells as template. The two PCR fragments were separately inserted into pGL3-basic vector after digestion with restriction endonucleases *Nhe*I and *Hin*dIII, and the resulting plasmids were named as pLucM1 and pLucM2, respectively.

### Luciferase reporter assay

PLC and Huh7 cells were seeded in 48-well plates and were co-transfected with pLucM1 or pLucM2 and monitor plasmid pCMV-β-gal using Lipofectamin 2000 according to the manufacturer’s protocol. After 36 h, the cells were lysed, and luciferase activity and β-gal activity were separately detected using Promega luciferase and β-gal assay systems according to the manufacturer’s protocols. The luciferase activity was normalized against β-gal activity. The transfection experiments were performed at least three times in triplicate. Data were represented as fold induction by normalizing the luciferase activity of the tested sample to that of the corresponding control sample.

### Trypan blue exclusion assay

The trypan blue exclusion assay was performed as described [41]. The total death rate (%) = numbers of dead cells/(numbers of living cells + numbers of dead cells) × 100.

### Flow cytometry

After treatment, the HCC cells were harvested and incubated with annexin V-FITC and PI according to the manufacturer’s instructions. Then the apoptosis were analyzed by a flow cytometer.

### Statistic analysis

The data were expressed as Mean ± SD. Two-way *t*-test and ANOVA were used to analyze the variance. *P* < 0.05 was defined as statistically significant.

## Competing interests

The authors declare that they have no competing interests.

## Authors’ contributions

BW and Z-HN mainly performed the Western blot tests, data collection, data analysis and the draft of this manuscript. X-FD and L-YQ mainly performed the functional assays. X-ZL performed the luciferase assays. LX contributed to the discussion section and interpretation of some results. J-QL and F-TH contributed to the study design and the manuscript revision. All authors read and approved the final manuscript.

## Supplementary Material

Additional file 1: Figure S1 Bcl-2 inhibitor AT-101 upregulates Mcl-1 in four HCC cell lines. HCC cells were treated with 10 μM AT-101 or vehicle DMSO for 18 h, then the protein level of Mcl-1 was analyzed by Western blot, taking α-tubulin as a loading control. **Figure S2.** N-acetyl-L-cysteine dramatically attenuates ABT-263-induced Mcl-1 upregulation and phophorylation of ERK, JNK and Akt. After pretreated with 10 mM N-acetyl-cysteine (NAC) for 2 h, the HCC cells were treated with 5 μM ABT-263 or vehicle DMSO for another 18 h. Then Mcl-1, p-ERK, p-JNK and p-Akt were detected by Western blot, taking α-tubulin as a loading control. Click here for file
