# Supplementary figures and images for: The Bcl-2/xL inhibitor ABT-263 increases the stability of Mcl-1 mRNA and protein in hepatocellular carcinoma cells
Source: Mol Cancer. 2014 Apr 30;13:98. doi: 10.1186/1476-4598-13-98 (PMC4021276; doi:10.1186/1476-4598-13-98)

# Supplementary Figure 1

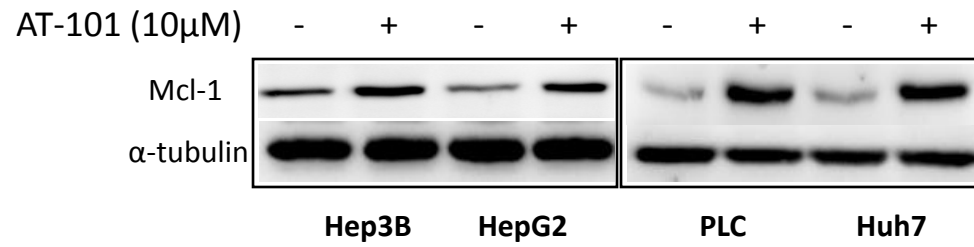

## Supplementary Figure 2

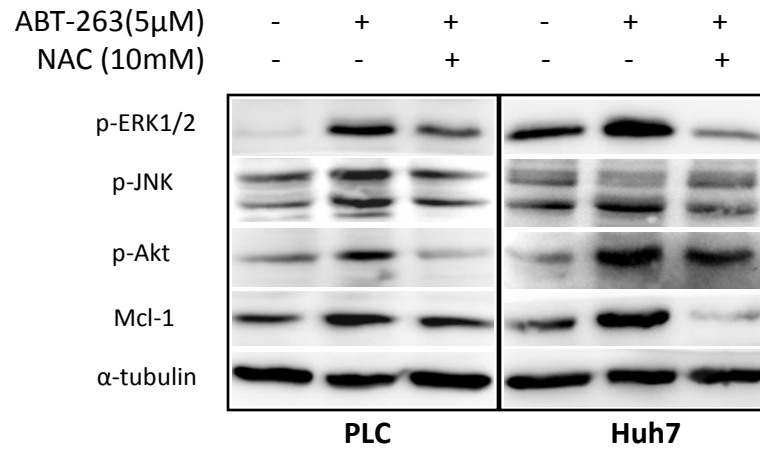

Supplement: Additional file 1: Figure S1 — Bcl-2 inhibitor AT-101 upregulates Mcl-1 in four HCC cell lines. HCC cells were treated with 10 μM AT-101 or vehicle DMSO for 18 h, then the protein level of Mcl-1 was analyzed by Western blot, taking α-tubulin as a loading control. Figure S2. N-acetyl-L-cysteine dramatically attenuates ABT-263-induced Mcl-1 upregulation and phophorylation of ERK, JNK and Akt. After pretreated with 10 mM N-acetyl-cysteine (NAC) for 2 h, the HCC cells were treated with 5 μM ABT-263 or vehicle DMSO for another 18 h. Then Mcl-1, p-ERK, p-JNK and p-Akt were detected by Western blot, taking α-tubulin as a loading control. [file 1476-4598-13-98-S1.pdf]
